# In-depth analysis of sampling practice and yield of blood cultures

**DOI:** 10.1007/s10096-026-05568-4

**Published:** 2026-06-25

**Authors:** Thea Kippersund Rønningen, Frode Width Gran, Jan Kristian Damås, Jan Egil Afset, Birgitta Ehrnström

**Affiliations:** 1 Faculty of Medicine and Health Sciences, Norwegian University of Science and Technology (NTNU), Trondheim, Norway; 2 Department of Medical Microbiology, St. Olav’s Hospital, Trondheim University Hospital, Trondheim, Norway; 3 Central Norway Sepsis Research Centre, Department of Clinical and Molecular Medicine, Faculty of Medicine and Health Sciences, Norwegian University of Science and Technology (NTNU), Trondheim, Norway; 4 Clinic of Anaesthesia and Intensive Care, St. Olav’s Hospital, Trondheim University Hospital, Trondheim, Norway; 5 Department of Infectious Diseases, Clinic of Internal Medicine, St. Olav’s Hospital, Trondheim University Hospital, Trondheim, Norway

**Keywords:** Blood stream infections, Blood culture, Detection of microorganisms, Sample volume, Optimising results, Clinical relevance

## Abstract

**Purpose:**

To describe BC collection practices at a tertiary care university hospital in a prospective observational study and examine the association between blood volume and culture yield.

**Methods:**

Analysis of all blood cultures collected during a 6-week period in 2023 at St Olav’s University Hospital, Norway. Additionally, we analysed impact of antimicrobial treatment prior to sampling, sampling department, bottle type, sampling time and method, microorganisms isolated as well as patient age and sex.

**Results:**

Only 19.7% of BC bottles were filled with the recommended 8–10 mL of blood. The overall pathogen positivity rate of all BC bottles was 6.8%, while 13.3% of the patients had at least one positive BC bottle. Patients >60 years were twice as likely to have a positive blood culture as those aged 18–60 years (16.2% vs 8.1%). Recommended filling volumes were positively associated with recovery of microbes, whereas particularly overfilling >14 mL was associated with reduced microbial yield. Overall, bottles with no growth contained a significantly higher mean blood volume than bottles with growth.

**Conclusion:**

This study highlights the gap between established recommendations for BC collection and current practice at our hospital. Preanalytical optimisation of BC collection is needed to improve diagnostic yield in this seriously ill patient population. Overfilling of BC bottles was negatively associated with microbial yield and should be avoided.

**Supplementary Information:**

The online version contains supplementary material available at https://doi.org/10.1007/s10096-026-05568-4.

## Introduction

Bloodstream infections (BSIs) constitute a public health concern due to the major impact on morbidity and mortality of the general population [1–3]. There are also reports on increased incidence of BSIs over the past decades [4–7]. The diagnosis of BSIs relies on detection of pathogens in blood. Major achievements have been made the past decades to improve identification of microorganisms via molecular rapid diagnostic testing [8], with a particular focus on reducing time to identification. Despite the availability of novel test methodologies such as amplification, hybridization, and mass spectrometry methods, blood culture (BC) is still considered the gold standard for detection of the infecting agent in BSIs. In addition, BC allows for phenotypic susceptibility testing of the pathogen.

Blood culture yield is influenced by several factors, of which the most important are regarded blood volume per bottle, the number of blood culture sets collected, BC collection before initiation of antimicrobial treatment, and sampling site [9–13]. In BSIs, the concentration of microorganisms in blood is typically low, usually less than 10 colony forming units (CFU)/mL and in 50% of BSI episodes 0,01–1 CFU/mL [12, 14]. Culture of sufficient blood volume has been identified as the single most important variable for detection of pathogens [13–17]. It is recommended that abundant volume of blood is sampled, usually 40–60 mL divided into 4–6 bottles for adult patients [9, 18, 19]. The optimal volume per bottle is considered 8–10 mL and the route should be peripheral venipuncture [20]. Blood drawn via syringe, venous catheter or arterial catheter is not recommended due to increased risk of contamination [21, 22].

The sensitivity of BC increases from 65 to 75.7% to 80.4–89.2% and 95.7–97.7%, respectively, when 20, 40 and 60 mL of blood is collected [10, 13, 23, 24]. Neves et al. found that blood volume was a significant predictor of positivity with an odds ratio of 1.01 i.e. each additional mL of blood gave an increase of 1% in positivity [25]. Despite this, there are numerous publications reporting that underfilling bottles is common [26–31].

The objective of this observational study was to conduct a prospective analysis of blood volume distribution and the relationship between sampled volume and yield of blood cultures at St Olav’s University Hospital in Trondheim, Norway. Additionally, we investigated the effect on BC yield of antimicrobial treatment prior to sampling, bottle type, department, sampling time and method, the microorganisms isolated as well as age and gender of the patient.

## Materials and methods

### Study setting

This prospective study included the BCs collected by venipuncture and venous or arterial catheters from patients at St Olav’s University Hospital departments in Trondheim during six consecutive weeks in February and March 2023. Common practice is that BCs are collected by nurses and only occasionally by dedicated phlebotomists when BCs are particularly difficult to obtain. The BC bottles were transferred to the microbiological department at the same hospital according to standard procedure. St Olav’s University Hospital has a total of approximately 1000 somatic beds in the departments situated in Trondheim. Only BC bottles from patients ≥ 18 years of age were included.

### Data collection/blood cultures

For each bottle the following parameters were registered: bottle type, blood volume, sampling department, antimicrobial treatment prior to sampling, sampling time, time of arrival of BC bottles at the microbiology laboratory, the microorganisms isolated as well as age and sex of the patient. All individual BC bottles were weighed with an accuracy of +/- 0.1 gm on an electronic scale. The blood volume in each bottle was calculated using the following formula: (weight of bottle filled with blood − mean weight of an empty bottle)/density of blood (1.055 g/mL) [13, 26]. The bottles were produced by BD: BD BACTEC Plus Aerobic/F, BD BACTEC Lytic/10 Anaerobic/F and BD BACTEC Mycosis IC/F. 50 empty bottles of each type were weighed, and mean weight was calculated. The mean weight was 63.9 (range 63.4–64.9), 60.2 (59.8–60.9) and 65.2 (65.1–65.3) gm for mycosis, aerobic and anaerobic bottles, respectively. Bottles with a positive culture signal were further tested with Matrix-assisted laser desorption ionization-time of flight mass spectrometry (MALDI-TOF MS) to identify the microorganisms on a Maldi Biotyper software, with MBT Compass Library DB-10,833 MSP (Bruker Daltonics). Culture positive bottles were weighed when taken out of the incubator due to the generation of a positive signal, whereas the negative bottles were weighed when removed from the incubator after seven days. The BCs were incubated in a BD Bactec FX instrument (Becton Dickinson, USA). Data was retrieved from the laboratory information system (LIS) using the individual bottle instrument number. Also related information, such as patient ID, age, sex, and department, was extracted from LIS.

### Definitions

One pair of aerobic + anaerobic bottles collected from the same sampling site constituted a blood culture set. Negative bottles were defined as bottles with no growth after seven days of incubation. Positive bottles were defined as bottles with growth of any microorganisms, including those considered contaminants, within seven days of incubation. These were then further categorized into bottles with growth of clinically relevant microorganisms and bottles with a likely contaminant.

### Deduplication period

A BSI episode was defined as an episode where one or more relevant microorganisms were discovered in one or more BC bottles sampled. A new episode was recorded if a different bacterial or fungal species was detected in BCs collected from the same patient more than 72 h after the first positive blood culture was collected. If the same microbe was found in a BC from the same patient again ≥ 30 days after a first episode, this was also considered a new episode. A polymicrobial episode was defined when more than one bacterial species was detected in BC collected within a period of 72 h.

### Determination of contaminants

We classified the following bacteria as contaminants: coagulase-negative staphylococci, *Bacillus* spp. (not anthracis), *Cutibacterium acnes*, *Corynebacterium* spp., and *Micrococcus* spp [12, 19, 26, 32, 33]. The bacteria mentioned above rarely represent a clinical infection, and detailed knowledge of the patient’s clinical condition would have been necessary to determine a possible clinical significance. This was not within the scope of the present study.

### Statistics

Statistical analyses were made with IBM SPSS Statistics 29.0.0.0. Distribution of blood volume in the BC bottles: normality was evaluated using a histogram and a Q–Q plot, which showed no major deviations from a normal distribution aside from a mild right skew. Kolmogorov–Smirnov and Shapiro–Wilk tests were also performed to support this assessment. The Chi-square test and analysis of variance were used to compare separate pair groups and the overall recovery difference between the volume groups. The various volume analyses are presented as mean with 95% confidence interval. A p value of < 0.05 was considered statistically significant.

### Ethics

This study was conducted in accordance with the Declaration of Helsinki. The regional ethical committee has assessed the project (527192/2022). The Regional Committee for Medical and Health Research Ethics (REK) assessed this project as a quality control project. Consent from patients was not needed. Sensitive patient data were stored securely at St Olav’s University Hospital data server, and upon completion of data collection, each patient was assigned a study number for deidentification. The key between the personal identification and study numbers was stored in a separate folder. A data protection impact assessment was conducted and approved by head of Clinic of Laboratory Medicine.

## Results

In total 4020 individual bottles from 775 patients were received and registered at the microbiology laboratory during the six-week study period. Of these, 39 bottles were not weighed and therefore excluded from the analysis with volume as a parameter. Four additional bottles were excluded from the same analysis due to negative blood volume after conversion from weight to volume. All sampled BC bottles collected constituted a part of a set (one aerobic and one anaerobic bottle). For 162 of the patients (20.9%), one single set of BC bottles was obtained within the first 24 h after the first BC set was collected.

An overview of the characteristics of patients, BC bottles and hospital departments included in the study is presented in Table 1. Almost half of the BCs were collected at the Emergency Department (45.5%) (Table 1). BCs were more often collected from male than female patients (61.0 vs. 39.0%) and contributed to a higher percentage of positive BCs (59.2% vs. 40.8%)(Supplementary table 1A). 64.8% of the BCs were drawn from patients above 60 years of age and 78.6% of all positive bottles were sampled from this age group (Supplementary Table 2). In total, 13.3% of the patients had at least one positive BC bottle with growth, including both microorganisms considered clinically relevant and those considered contaminants. Patients older than 60 years were twice as likely to have a positive blood culture (16.2%) compared to patients between 18 and 60 years (8.1%).

**Table 1:** Overview of the characteristics of the blood culture bottles and patients included

	Number of bottles (% of total)
All bottles analysed	3977 (100)
Male	2177 (54.7)
Female	1800 (45.3)
With growth of microorganisms	270 (6.8)
No growth*	3707 (93.2)
Bottle type	
Aerobic	1931 (48.6)
Anaerobic	1920 (48.3)
Mycosis	126 (3.2)
Hospital department	
Emergency department	1808 (45.5)
Surgical departments	298 (7.5)
Medical departments	909 (22.9)
Department of Oncology	528 (13.3)
Intensive Care Unit	183 (4.6)
Department of Neurology	205 (5.2)
Department of Gynecology	46 (1.2)

*Including 6 bottles that signalled positive in the incubator, but resulted in no findings on MALDI-TOF MS. Five of these bottles were overfilled

### Blood volume

**Fig. 1:**
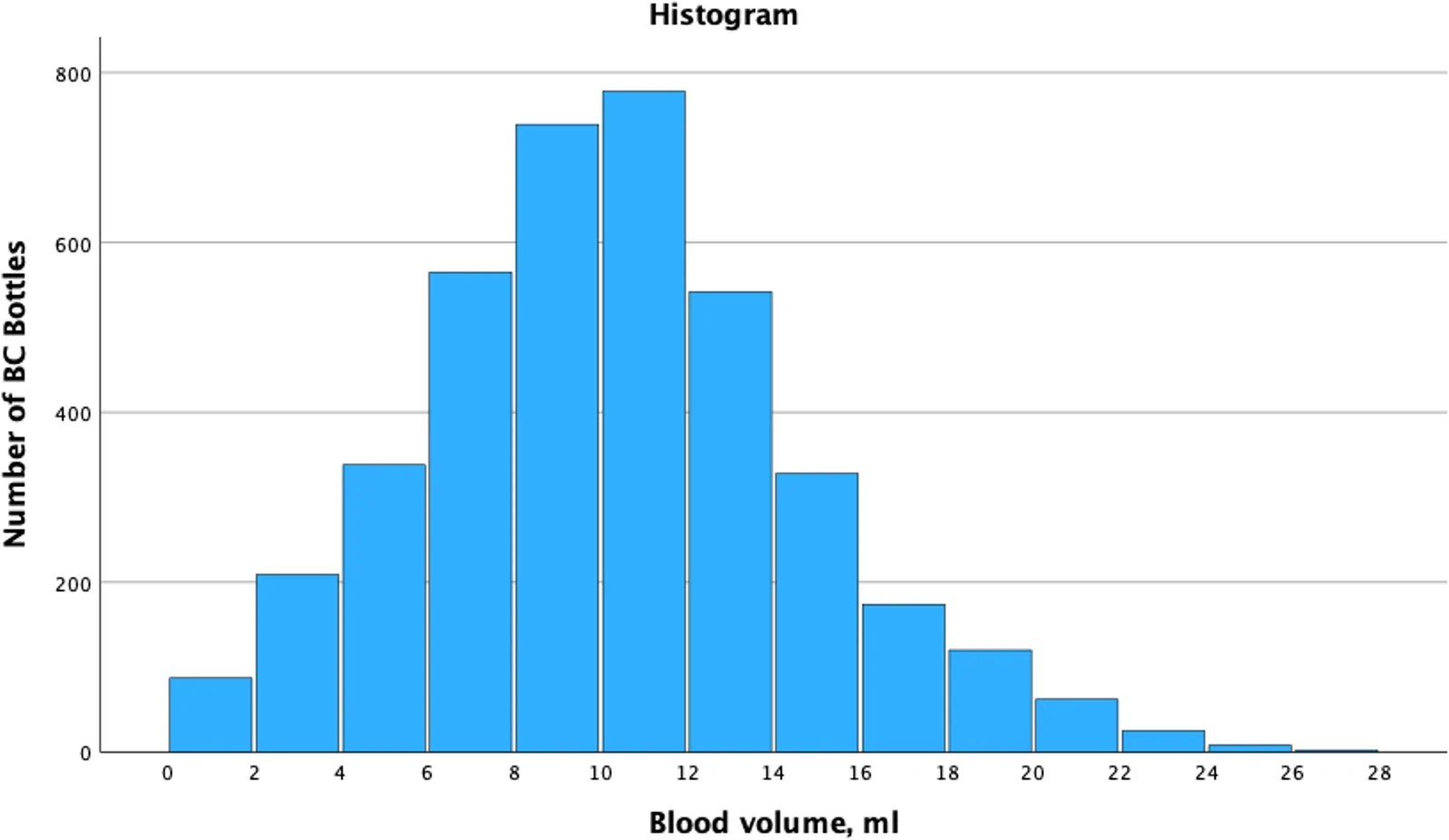
Histogram presenting number of BC bottles per collected blood volume in 2-mL classes. *n =* 3977 BC bottles

**Fig. 2:**
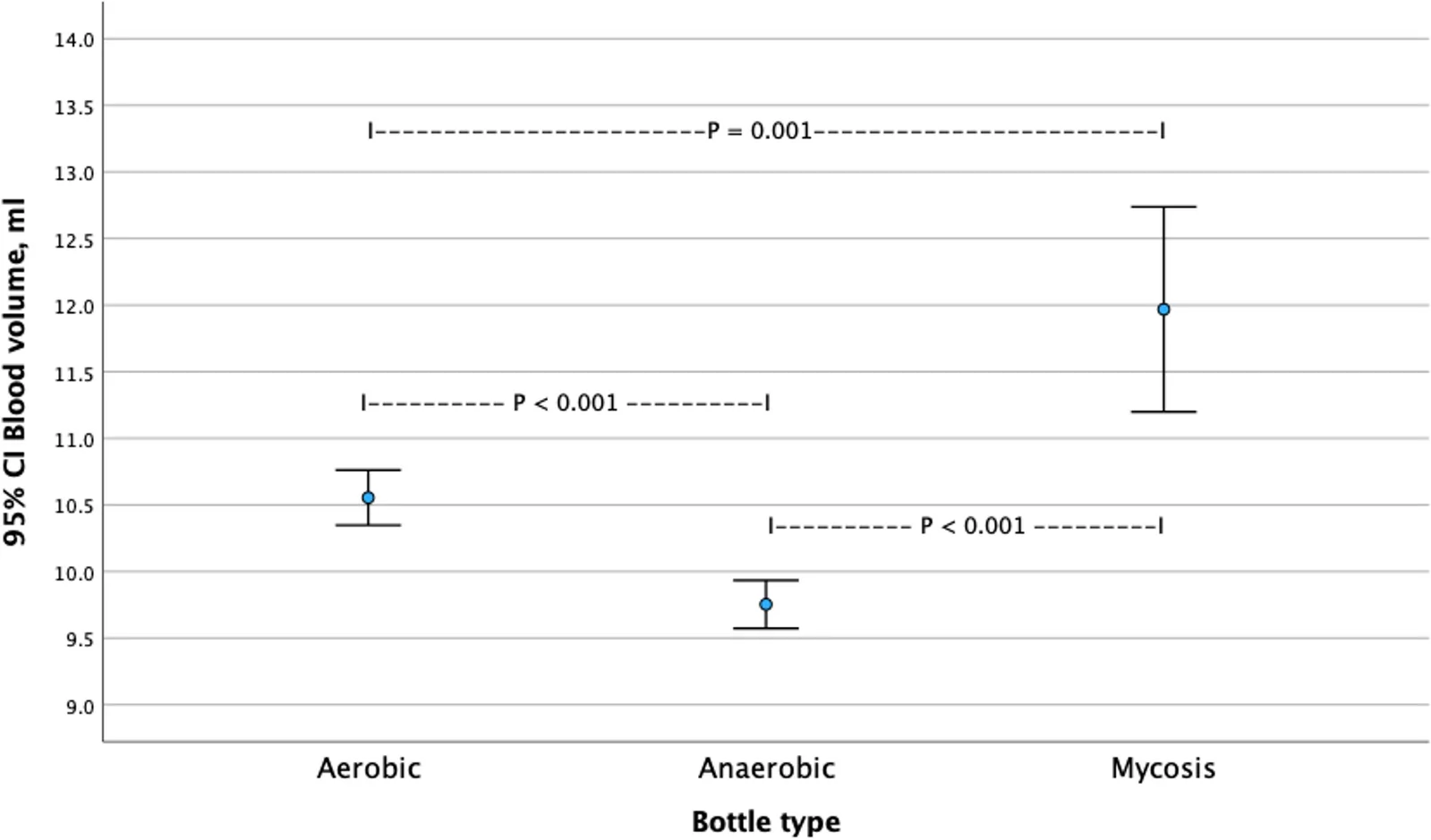
Volume of blood collected in aerobic, anaerobic and mycosis BC bottles shown as mean with 95% confidence interval (CI)

Only 785 bottles (19.7%) were filled with the recommended volume of 8–10 mL, and the remaining bottles were filled with < 8 mL (*n* = 1199, (30.2%)) or > 10 mL of blood (*n* = 1993, (50.1%)). Figure 1 shows the distribution of sampled blood volume (range 0.1–27.0 mL blood). There were significant differences in mean blood volume between all the three different BC bottle types (Fig. 2). Mycosis bottles had the highest mean blood volume (± standard deviation) with 11.97 (± 4.37) mL, followed by aerobic bottles with 10.55 (± 4.63) and anaerobic bottles with 9.75 (± 4.03) mL. Only 17.4% of the aerobic bottles were filled with the recommended volume of 8–10 mL. There were no significant correlations between patient age and BC bottle content of blood volume.

### Correlation between blood volume and positive BC rate

**Fig. 3:**
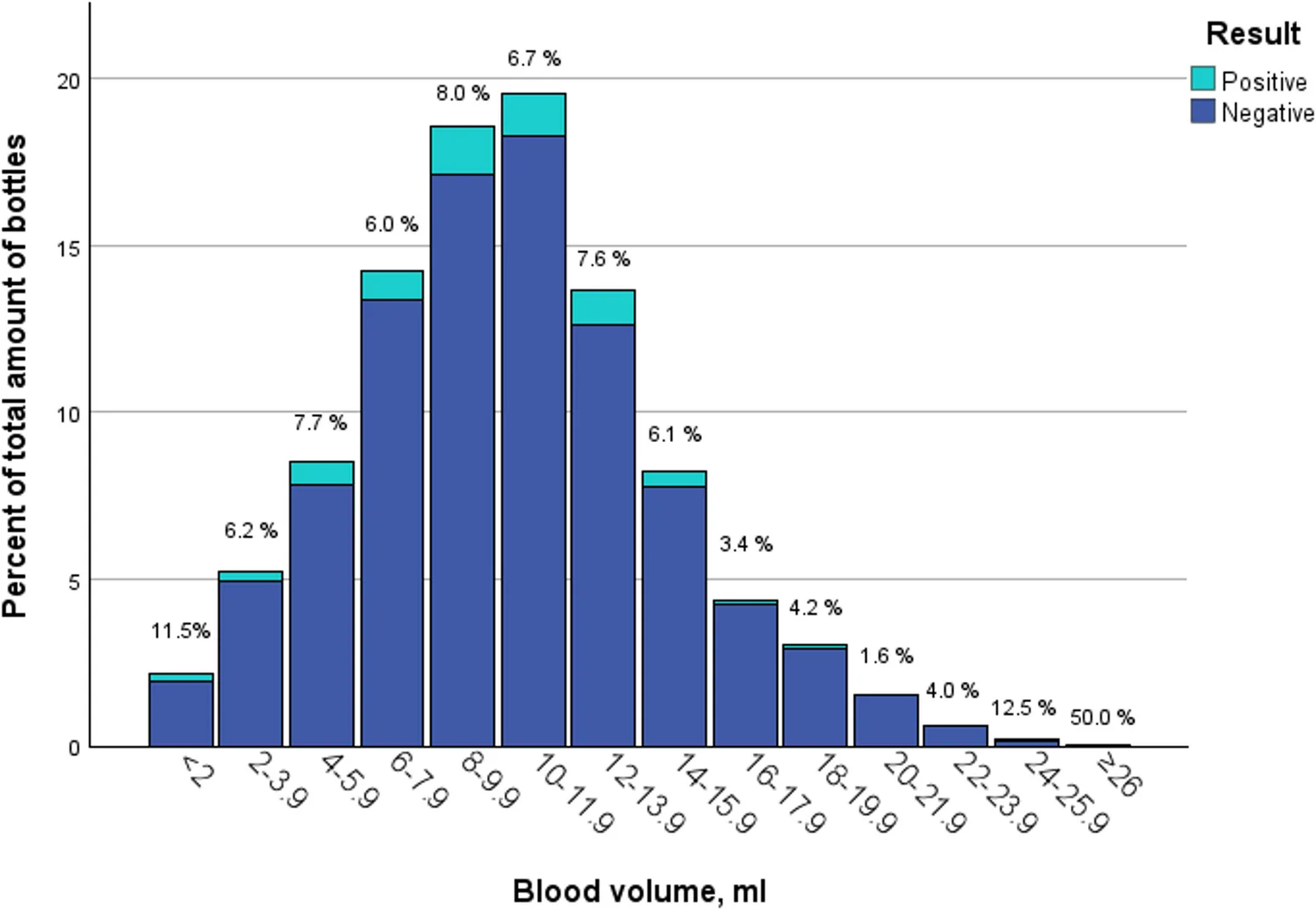
Proportion of the total amount of BC bottles that are culture positive. Above each column is the percentage of bottles with positive yield in the respective volume interval noted

**Fig. 4:**
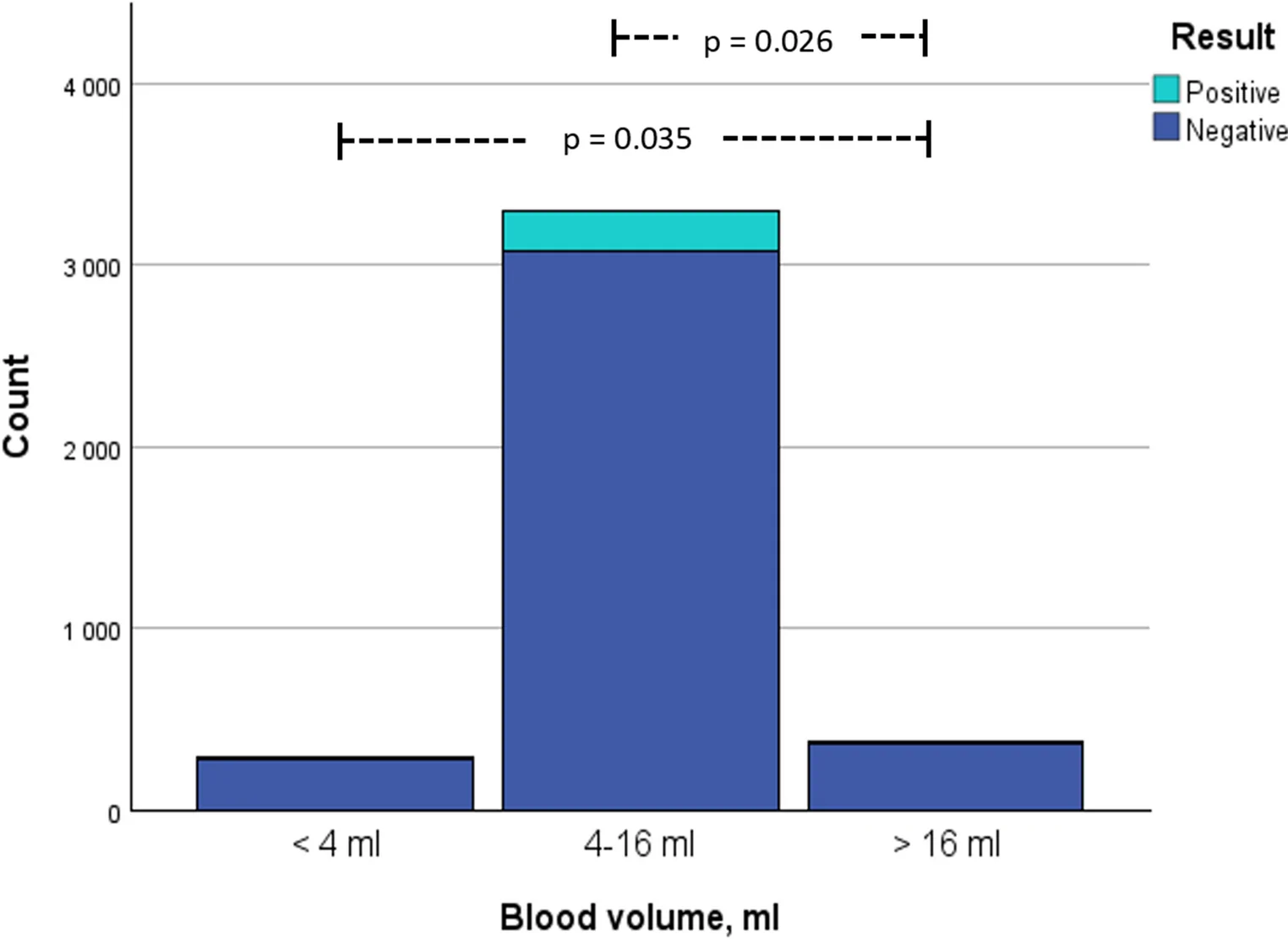
Blood volume in BC bottles divided into three volume classes. Statistically significant findings are marked with p-value. There was a microbial yield in 23/273 of the bottles containing < 4 mL blood, in 232/3072 of the bottles containing 4–16 mL blood and in 15/361 of bottles containing more than 16 mL blood. The positivity of BC bottles containing very low volume (< 4 mL, *n* = 296) or intermediate volume (4–16, *n* = 3305) mL were significantly higher than for bottles containing very high blood volume (> 16 mL, *n* = 376), *p* = 0.026 and *p* = 0.035, respectively

The impact of blood culture volume was analysed by assessing positivity rate (Fig. 3). Growth of clinically relevant microorganisms was detected in 270 of 3977 BC bottles (6.8%). The highest yield was found in aerobic bottles (149/1932, 7.7%), followed by anaerobic bottles (121/1920, 6.3%), while there was no growth in any of the 126 mycosis bottles. There was no statistically significant difference in positivity rate between BCs with 8–10 mL of sampled blood compared to the BC bottles with other volumes including < 8 and > 10 mL blood. To evaluate the effect of very high and low blood volumes, we compared the results between < 4 mL and > 16 mL blood against a broad intermediate volume group of 4–16 mL. The positivity rate in the intermediate volume group (4–16 mL, *n* = 3305) mL was significantly higher than for bottles containing very high blood volume (> 16 mL, *n* = 376) (*p* = 0.026). There was also a significantly lower yield in bottles filled with very high volume compared to those containing less than 4 mL (*n* = 296) (*p* = 0.035) (Fig. 4). And bottles with no growth contained a significantly (*p* = 0.047) higher mean volume of blood than bottles with growth, 10.25 (± 4.38) mL and 9.70 (± 4.22) mL, respectively.

### Sample volume distribution between departments

**Fig. 5:**
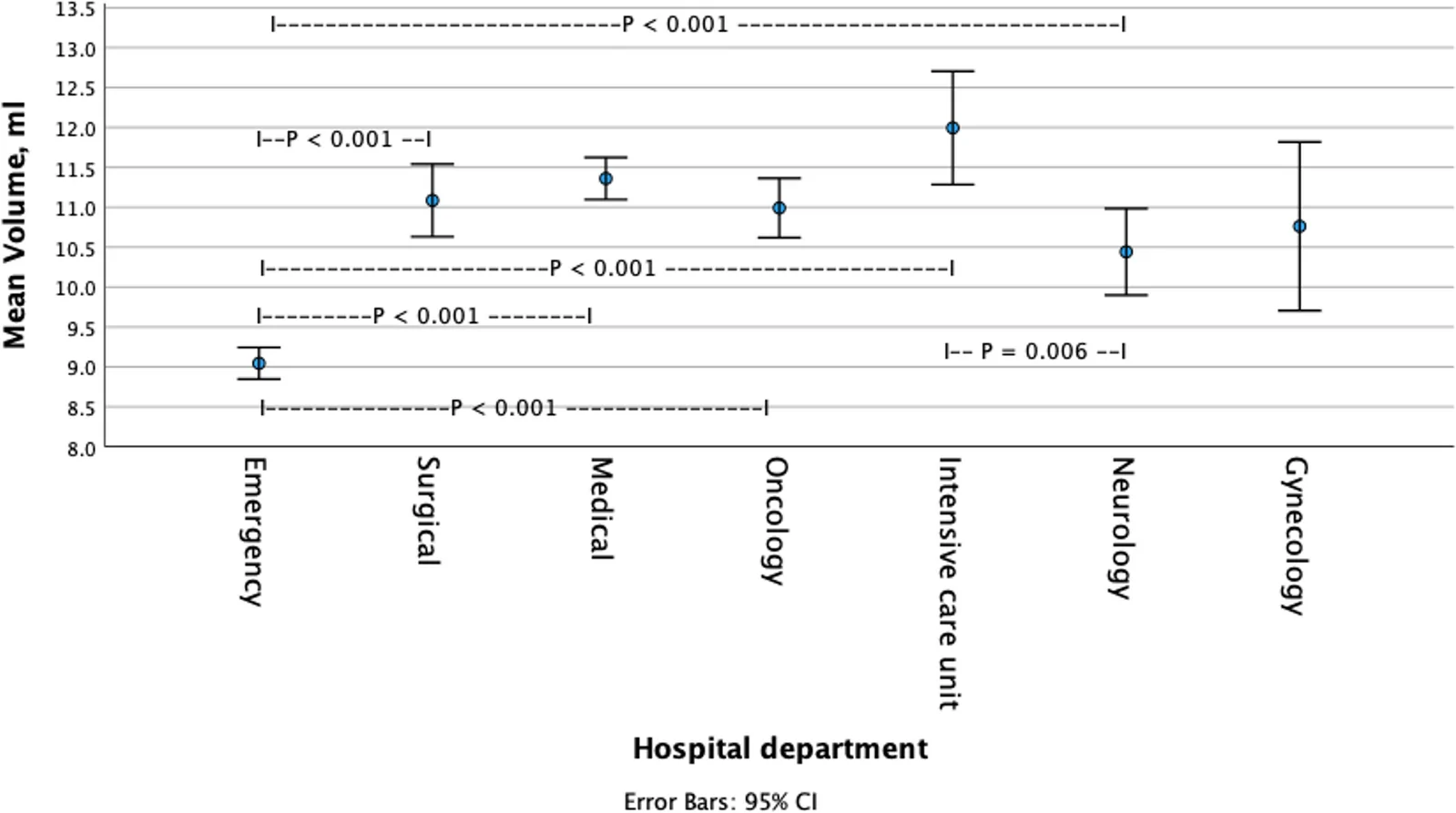
Volume of blood collected in bottles from different hospital department, shown as mean with 95% confidence interval (CI). Statistically significant findings are marked with p-value. The mean blood volume in mL (± standard deviation) of the departments from the left, in the presented order, was 9.04 (± 4.31), 11.08 (± 3.99), 11.36 (± 4.04), 10.99 (± 4.35), 11.99 (± 4.87), 10.44 (± 3.93) and 10.76 (± 4.55)

A comparison of BC bottles with respect to the collecting hospital department was performed, showing that only the Emergency department had a mean sample blood volume within the recommended interval 8–10 mL. There was a statistically significant difference in mean blood volume sampled at the Emergency Department, compared to all other departments (*P*-value < 0.001) except the Dept. of Gynecology (Fig. 5).

### Sample method

The blood volumes sampled from arterial tap and central venous catheter (CVC) were significantly higher compared to sampling performed by direct venipuncture (*P* < 0.001). The mean blood volume collected (± standard deviation) was 9. 90 (± 4.26) mL from bottles sampled by peripheral venipuncture, 12.35 (± 4.61) mL sampled by arterial tap, 12.17 ± 4.45 mL sampled by CVC and 11.07 (± 4.72) mL when the sampling site was not specified. In total, 3410 (85.7%) of the BC bottles collected were sampled by peripheral venipuncture.

### Blood culture positivity and antibiotic treatment

There was a significant difference in positivity rate with regard to whether antibiotic treatment had been commenced before or after BC collection (*P* < 0.001). Growth of microorganisms was detected in 9.0% of BC bottles from patients where antimicrobial therapy had not been started before BC collection (Supplementary Fig. 1), compared to only 2.8% of BC bottles from patients already on antibiotic treatment. Microorganisms classified as clinically relevant were detected in 280 BC bottles (123 Gram-negative, 156 Gram-positive and 1 *Candida* sp.) in 89 patients, while microorganisms classified as contaminants were detected in 1.1% of the BC bottles (45 bottles from 37 patients) (Supplementary Tables 2 and 3). There were 26 bottles with polymicrobial growth when contaminants were included. *Escherichia coli* (28.2%) and *Staphylococcus aureus* (18.8%) were the most frequent findings.

### Analysis with no significant findings

Blood cultures were collected through day and night; 1607 (40.4%) BC bottles were collected during daytime (07:30 am – 3:29 pm), 1474 (37.1%) during afternoon or evening (3:30 pm – 09:59 pm), and 896 (22.2%) during nighttime (10:00 pm – 07:29 am). There were no significant differences in the average blood volume between the time intervals in which the BC bottles were sampled. Neither were there any significant gender differences observed with regard to blood volume sampled, although bottles from male patients had a somewhat higher mean blood volume (± SD) than bottles from female patients, 10.30 (± 4.48) and 10.10 (± 4.24) mL respectively. The bottles with clinically relevant microbes contained a higher mean volume of blood (± SD) collected, 9.78 (± 4.37) mL, than the bottles with contaminants (9.29 (± 3.14) mL), though this difference was not statistically significant.

## Discussion

The present study reveals that the proportion of BC bottles with optimal blood volume was low; only one fifth of the bottles were filled with the recommended volume 8–10 mL blood. Overfilling was observed in 50% of the bottles and interestingly, volumes above 14 mL were associated with decreased recovery rate. Further, we observed that BC bottles sampled from older patients were twice as likely to yield growth than younger patients. 13.3% of the patients had at least one positive BC bottle. There were both similarities and differences when comparing to other previously published observations.

Bacteremia may be transient and the number of bacteria/mL during BSI is low [12]. Several studies have observed that blood volume is the most important factor for recovery of pathogens from blood [10, 13, 23–25, 34]. Inadequate sampling is a hazard to successful diagnosis and treatment of patients with BSI. The sampling of blood cultures includes several elements.

### Degree of filling

In our study, only 19.7% of the bottles were filled with the recommended volume. This is in line with other publications, though considerable variations are reported [26–31]. A recent study from Sweden found that only 18.2% of the BC bottles contained an adequate blood volume and 47% less than < 8 mL of blood [26]. But in contrast to most other studies, we found that both underfilling (30.2%) and overfilling (50.1%) was common in our study. A Belgian multi-center study reported that 7.6%–12.8% of the bottles were overfilled, whereas in 26.3–36.0% the volume was below the recommended level [29]. As a quality indicator it has been proposed that > 80% of all adult aerobic BC bottles should contain 8–10 mL of blood [9, 12]. Low sample volume increases the risk to underdetect BSIs since the concentration of microbes in blood often is very low. Our results confirm this, where we found a higher positivity rate among bottles containing 8–10 mL of blood than those with a lower blood volume.

On the other hand, overfilled bottles risk generating a falsely positive signal by the BC system. The absolute number of leukocytes in a BC bottle has been shown to be the most discriminating factor of early false positivity [35]. Overfilling also results in blood-to-broth ratios above recommended levels, with a risk of decreased microbial recovery and false-negative result. This phenomenon has been attributed to the inhibitory effect of complement, phagocytes and antimicrobial agents but is less frequently addressed [16, 30]. Indeed, we observed a trend of decreased recovery rate for blood volumes above 14 mL with significantly lower positivity rates for blood volumes above 16 mL compared to lower volumes. This finding equate observations by Lin et al. who saw a positive correlation between volume and recovery rate up to 10 ml, but a decrease in recovery rate above 10 ml per bottle [30]. At least in our setting with half of all BC bottles being overfilled, this poses a clinically relevant problem for diagnosing BSI. Additionally, overfilling of BC bottles may induce phlebotomy-related anemia. One reason for the general problem with inadequately filled BC bottles may be the lack of correlation between the vacuum in the bottles and the optimal blood volume that needs to be collected [29]. A way to overcome inadequate filling and other issues with sampling may be dedicated phlebotomy teams that have been reported to be highly cost-effective by reducing contamination rates, increasing true positive rate and optimizing blood volumes [28, 36]. A recent systematic review and meta-analysis by the American Society for Microbiology identified several interventions that resulted in significant reduction of BC contamination: chlorhexidine as skin disinfectant, use of diversion device, sterile technique practices, phlebotomy teams, and education programs [37, 38].

### Number of BC bottles

In our material, only one single set of BC was collected from 20.9% of the patients, which is approximately twice the proportion recommended as the minimum standard [12]. Other centers report a huge variation in proportion of single BC sets collected - between 3.4% and 42.5% [25, 39, 40]. Possible explanations in our material might be a low level of suspected BSI or that another set was planned later but forgotten. The high rate of single BC sets collected and corresponding lower total blood volume harvested, may have reduced the number of pathogens retrieved and thereby the recovery rate. Further, it has likely made it more difficult to accurately assess possible contamination.

### Blood volume/infection episode

Here, we report growth in 6.8% of all BC bottles, which may be considered somewhat low but still within the suggested ideal range. The other Scandinavian study reported a higher positivity rate (8.99%), and a lower mean number of bottles collected per patient than we did (mean 3.94 vs. 5.18 bottles) [26]. The American Society of Microbiology (ASM) has proposed that overall, an adequate positive rate of blood culture is 6–12% in any given center [41]. They state that a higher positivity rate (> 12%) might suggest that too few blood cultures are collected, and conversely a positivity rate less than 6% that too many BCs are sampled.

### Sampling method

85.7% of the BC bottles in this study were sampled by peripheral venipuncture, which is the recommended site to reduce the risk of contamination [21, 22, 42]. A meta-analysis found that venipuncture strongly reduced contamination rates compared to collection of catheter blood (OR 2.69, 95% CI: 2.03–3.57) [43]. The contamination rate in our material was 1.1%, which is close to the new quality measure of ≤ 1% [44]. Devices that divert the initial aliquot of blood can also reduce BC contamination rates considerably, by up to 80–90% [45, 46]. In our study, the mean volume from bottles sampled by arterial tap and central venous catheter (CVC) was higher than recommended. The majority of the collection by either of these two methods was from the ICU unit that also was the department with the highest mean sample volume.

### Culture yield

Growth of microorganisms were detected in 9% of BC bottles from patients not yet receiving antibiotic therapy, whereas the recovery rate was only 2,8% in BC bottles from patients on antibiotic treatment. Though this finding is not surprising, it highlights the importance of sampling blood culture before onset of antibiotic treatment. Administration of antibiotics prior to blood collection is the major critical factor impeding yield of culture. We argue that molecular rapid diagnostic testing can play a particularly important role here, as an alternative or complement to traditional culture of blood.

### Volume – age and gender

we observed no significant correlations between age and volume. However, the mean volume of sampled blood showed a decreasing trend from younger to older age patients. This trend is similar to the results in another Scandinavian study [26]. The same study observed that significantly larger blood volumes were collected from male than female patients. We observed the same trend, though not statistically significant. 2/3 of the blood cultures were drawn from patients above 60 years of age and were twice as likely to yield growth than those from patients below 60. These are expected results since BSI and sepsis are more frequent among the elderly, and consistent with other reports [47, 48]. There were no significant differences between the time of day the BC bottles were sampled and the volume of blood in the bottles – in contrast to findings by others [26].

### Limitations

There are some limitations to this study. We have no detailed clinical data on the patients. For more than 25% of the patients, we lack information on whether the patient received antimicrobial treatment at the time of BC collection. Another limitation is that the study was performed at a single hospital. We don’t have a large enough number of positive findings to perform some statistical analyses; an example is the small number of fungal microbes detected in our data set.

### Conclusions

Blood cultures remain the first and most important element in diagnosing serious, invasive infections. The positivity rate in our material was within the suggested optimal range and we observed that positive yield was more likely in the older patients. We show that multiple factors regarding sampling routines are suboptimal and that the blood volume too often is inadequate and varies between departments. High blood volumes were negatively associated with microbial yield. Improving routines for sampling of blood culture before onset of antimicrobial treatment remains important. We conclude that more effort is needed to improve sampling routines. Increased surveillance, continuous education, diversion devices and introduction of a dedicated phlebotomy team certified for BC collection could be solutions. We plan to follow up our findings with interventions and a new, larger study especially focusing on issues surrounding overfilled bottles.

## Supplementary Information

Below is the link to the electronic supplementary material.

Supplementary Material 1

## Data Availability

Raw data will be made available on reasonable request to researchers from accredited research institutions, with any data that may risk loss of confidentiality redacted. Access to data will be limited to investigators who provide a methodologically sound proposal. To ensure compliance with the General Data Protection Regulation, data processing must be covered by the European Commission’s standard contractual clauses for the transfer of personal data, which must be signed by the data requesters. Proposals and requests for data access should be directed to the corresponding author.
