# Supplementary material for: In-depth analysis of sampling practice and yield of blood cultures
Source: Eur J Clin Microbiol Infect Dis. 2026 Jun 25;45(10):3027–36. doi: 10.1007/s10096-026-05568-4 (PMC13676665; doi:10.1007/s10096-026-05568-4)
Supplement: Supplementary file 1 [file 10096_2026_5568_MOESM1_ESM.docx]

**Supplementary Figure 1: Antibiotic treatment before sampling of blood culture.** The percentage above each column in the diagram represents the proportion of positive bottles for the respective category.

|  | **Number (%)** |
| --- | --- |
| **All patients** | 773 (100) |
| Male | 471 (61.0) |
| Female | 302 (39.0) |
| **Patients with positive BC (including contaminants)** | 103 (13.3) |
| Male | 61 (59.2) |
| Female | 42 (40.8) |
| **Patients with negative BC** | 670 (86.7) |
| Male | 410 (61.2) |
| Female | 260 (38.8) |

**Supplementary Table 1A: Overview of the study population from which BC bottles were weighed.**

|  | **All patients** | | **Patients with positive BC** | | |
| --- | --- | --- | --- | --- | --- |
| **Age groups (years)** | Number | **%** | Number | **%*** | **%**** |
| 18-30 | 64 | 8.3 | 5 | 4.9 | 7.8 |
| 31-40 | 70 | 9.1 | 6 | 5.8 | 8.6 |
| 41-50 | 46 | 6.0 | 2 | 1.9 | 4.3 |
| 51-60 | 92 | 11.9 | 9 | 8.7 | 9.8 |
| 61-70 | 138 | 17.9 | 22 | 21.4 | 15.9 |
| 71-80 | 209 | 27.0 | 34 | 33.0 | 16.3 |
| >80 | 154 | 19.9 | 25 | 24.3 | 16.2 |
| **SUM** | 773 | 100 | 103 | 100 | 13.3 |

*Proportion of all patients with positive BC
**Proportion of all patients with positive BC within age group

**Supplementary Table 1B: Overview/Distribution of study population by age**

| **Gram-negative bacteria** | | |
| --- | --- | --- |
|  | **Number of bottles**  **(% of positive bottles)** | **Number of patients** |
| **Aerobic bacteria** |  |  |
| *Escherichia coli* | 87 (28.2) | 25 |
| *Enterobacter cloacae complex* | 4 (1.3) | 1 |
| *Serratia marcescens* | 5 (1.6) | 1 |
| *Klebsiella aerogenes* | 4 (1.3) | 1 |
| *Klebsiella pneumoniae* | 13 (4.2) | 6 |
| *Other* ***^a^*** | 6 (1.9) | 4 |
| **Anaerobic bacteria** |  |  |
| *Bacteroides ovatus* | 2 (0.6) | 1 |
| *Fusobacterium mortiferum* | 2 (0.6) | 1 |
| **Gram-positive bacteria** | | |
| **Aerobic bacteria** |  |  |
| *Enterococcus faecalis* | 24 (7.8) | 9 |
| *Enterococcus faecium* | 8 (2.6) | 3 |
| *Other Enterococcus spp.****^b^*** | 9 (2.9) | 2 |
| *Staphylococcus aureus* | 58 (18.8) | 16 |
| *Streptococcus pyogenes* | 14 (4.5) | 5 |
| *Streptococcus anginosus* | 4 (1.3) | 1 |
| *Streptococcus agalactiae* | 12 (3.9) | 2 |
| *Streptococcus dysgalactiae* | 1 (0.3) | 1 |
| *Streptococcus pneumoniae* | 8 (2.6) | 2 |
| *Other Streptococcus spp.****^c^*** | 17 (5.5) | 6 |
| **Anaerobic bacteria** |  |  |
| *Clostridium perfringens* | 1 (0.3) | 1 |
| **Fungi** | | |
| *Candida albicans* | 1 (0.3) | 1 |
| **^a^** *Aeromonas veronii, Haemophilus influenzae, Leclercia adecarboxylata, Raoultella ornithinolytica*  **^b^** Other Enterococcus spp.: *Enterococcus hirae, Enterococcus gallinarum*  **^c^** Other Streptococcus spp.: *Streptococcus mitis, Streptococcus parasanguinis, Streptococcus oralis, Streptococcus sanguinis* | | |

**Supplementary Table 2: Clinically relevant microorganisms isolated from blood cultures.**

| **Bacteria spp.** | **Number of bottles** | **% of bottles** | **Number of patients** |
| --- | --- | --- | --- |
| *Bacillus cereus* | 3 | 1.0 | 1 |
| *Cutibacterium acnes* | 4 | 1.3 | 4 |
| *Dermabacter hominis* | 1 | 0.3 | 1 |
| *Micrococcus luteus* | 2 | 0.6 | 2 |
| *Staphylococcus capitis* | 1 | 0.3 | 1 |
| *Staphylococcus epidermidis* | 12 | 3.9 | 9 |
| *Staphylococcus haemolyticus* | 1 | 0.3 | 1 |
| *Staphylococcus hominis* | 13 | 4.2 | 11 |
| *Staphylococcus simulans* | 1 | 0.3 | 1 |
| *Rothia mucilaginosa* | 1 | 0.3 | 1 |
| *Lacticaseibacillus rhamnosus* | 1 | 0.3 | 1 |
| *Pseudomonas stutzeri* | 2 | 0.6 | 1 |
| Bacteria (unspecified) | 3 | 1.0 | 3 |
| **Total** | 45 | 1.1 | 37 |

**Supplementary Table 3: Microorganisms isolated in blood cultures, categorised as contaminants.**
